# Neurofeedback-Linked Suppression of Cortical β Bursts Speeds Up Movement Initiation in Healthy Motor Control: A Double-Blind Sham-Controlled Study

**DOI:** 10.1523/JNEUROSCI.0208-20.2020

**Published:** 2020-05-13

**Authors:** Shenghong He, Claudia Everest-Phillips, Andrew Clouter, Peter Brown, Huiling Tan

**Affiliations:** ^1^MRC Brain Network Dynamics Unit at the University of Oxford, United Kingdom Oxford OX1 3TH; ^2^Nuffield Department of Clinical Neurosciences, University of Oxford, United Kingdom, Oxford OX3 9DU

**Keywords:** β burst, β oscillations, movement initiation, neurofeedback training

## Abstract

Abnormally increased β bursts in cortical-basal ganglia-thalamic circuits are associated with rigidity and bradykinesia in patients with Parkinson's disease. Increased β bursts detected in the motor cortex have also been associated with longer reaction times (RTs) in healthy participants. Here we further hypothesize that suppressing β bursts through neurofeedback training can improve motor performance in healthy subjects. We conducted a double-blind sham-controlled study on 20 human volunteers (10 females) using a sequential neurofeedback-behavior task with the neurofeedback reflecting the occurrence of β bursts over sensorimotor cortex quantified in real time. The results show that neurofeedback training helps healthy participants learn to volitionally suppress β bursts in the sensorimotor cortex, with training being accompanied by reduced RT in subsequent cued movements. These changes were only significant in the real feedback group but not in the sham group, confirming the effect of neurofeedback training over simple motor imagery. In addition, RTs correlated with the rate and accumulated duration of β bursts in the contralateral motor cortex before the go-cue, but not with averaged β power. The reduced RTs induced by neurofeedback training positively correlated with reduced β bursts across all tested hemispheres. These results strengthen the link between the occurrence of β bursts in the sensorimotor cortex before the go-cue and slowed movement initiation in healthy motor control. The results also highlight the potential benefit of neurofeedback training in facilitating voluntary suppression of β bursts to speed up movement initiation.

**SIGNIFICANCE STATEMENT** This double-blind sham-controlled study suggested that neurofeedback training can facilitate volitional suppression of β bursts in sensorimotor cortex in healthy motor control better than sham feedback. The training was accompanied by reduced reaction time (RT) in subsequent cued movements, and the reduced RT positively correlated with the level of reduction in cortical β bursts before the go-cue, but not with average β power. These results provide further evidence of a causal link between sensorimotor β bursts and movement initiation and suggest that neurofeedback training could potentially be used to train participants to speed up movement initiation.

## Abstract (precis)

Abnormally increased β bursts in cortical-basal ganglia-thalamic circuits are associated with rigidity and bradykinesia in patients with Parkinson's disease. Increased β bursts detected in the motor cortex have also been associated with longer reaction times (RTs) in healthy participants. Here we further hypothesize that suppressing β bursts through neurofeedback training can improve motor performance in healthy subjects.

## Introduction

β Band oscillations are a prominent feature in the cortical-basal ganglia motor network, and prove to be consistently modulated by movements and motor imagery when averaged over multiple trials (McFarland et al., 2000; Pfurtscheller et al., 2003). More recent studies have emphasized the importance of the temporal dynamics of the β oscillations and found that transient events of high-amplitude β oscillations (β bursts) in the cortical-basal ganglia motor network predict sensorimotor behavior (Feingold et al., 2015; Sherman et al., 2016). In particular, the rate of β bursts in the sensory cortex occurring close to the stimulus was more likely to impair perception (Shin et al., 2017). The timing of β bursts in the motor cortex before movement was related to the duration of motor preparation, with later bursts resulting in delayed response times (Little et al., 2019). Wessel (2020) also found that an increased rate of β bursts in the contralateral sensorimotor cortex following a go-cue predicted a longer reaction time (RT). However, all these studies are correlational, and it is still not clear whether modulating sensorimotor β bursts can lead to changes in motor preparation and movement initiation.

Neurofeedback training has been proposed as a promising technique to train patients to self-regulate pathologic brain activities to improve symptoms (Daly and Wolpaw, 2008; Erickson-Davis et al., 2012; Azarpaikan et al., 2014; Huster et al., 2014; Ros et al., 2014; Fukuma et al., 2018). It also offers the potential to provide more causal inference about manipulated brain states and their behavioral consequences. Khanna and Carmena (2017) trained healthy macaque monkeys to perform a sequential neurofeedback-motor task paradigm and showed that reaching movements preceded by a reduction in cortical β power achieved through neurofeedback training exhibited significantly faster movement onset times than those preceded by an increase in β power. Similarly, healthy human participants can also learn to voluntarily modulate β oscillations measured over the sensorimotor cortex with neurofeedback training (Vernon et al., 2003; Boulay et al., 2011; Witte et al., 2013). Combined behavioral tasks showed that increasing amplitude sensorimotor β rhythms were associated with longer RTs than decreasing amplitude sensorimotor β rhythms (Boulay et al., 2011; McFarland et al., 2015). However, there were large variations between participants in terms of the effect of the neurofeedback training (McFarland et al., 2015). In addition, there is a lack of proper sham control in most neurofeedback studies to determine whether veritable neurofeedback was the primary factor accounting for observed behavioral alterations, or whether other mental strategies were mediating the effects (Thibault et al., 2015, 2016). This is particularly relevant for neurofeedback studies targeting sensorimotor β oscillations since β activity can be reduced by motor imagery and attention (Pfurtscheller and Neuper, 2001; Vukelić et al., 2019). A clear association between neurofeedback training and enhanced performance both in terms of self-regulation of EEG β bursts and motor skills has yet to be established (Jeunet et al., 2019).

In this study, we conducted a double-blind sham-controlled study in healthy young participants using a sequential neurofeedback-behavior task with the visual feedback specifically indicating the occurrence of high amplitude β bursts measured from motor cortex. The results show that veritable neurofeedback training helped participants learn to suppress cortical β bursts better than sham feedback. The “real feedback,” but not “sham feedback,” training was accompanied by reduced RT in subsequently cued movements. Moreover, the RT of the motor task significantly correlated with the β burst characteristics (burst rate and accumulated duration) before the go-cue across all tested hemispheres, but not with the averaged β power. The changes in the accumulated β bursts before go-cue induced by the neurofeedback training correlated with the changes in subsequent RT. These results strengthen the link between cortical β bursts and movement initiation and further suggest that neurofeedback training could be a potential tool to train participants to speed up movement initiation in healthy participants and in patients with movement disorders such as Parkinson's disease.

## Materials and Methods

### Ethics

The present study was conducted in accordance with the Declaration of Helsinki, approved by local ethics committee, and all participants provided informed written consents before the experiments.

### Participants

Twenty young healthy participants aged 18–21 (10 females) were recruited for this study. The participants were pseudo-randomly assigned to a sham feedback group or a real feedback group, with ten participants in each group. The existence of a sham feedback group was blinded to all participants. Both the participants and the experimenter who gave instructions and conducted the recordings were blinded about the group each participant was assigned to, resulting in a double-blind sham-controlled design.

### Experimental protocol

**Figure 1.**
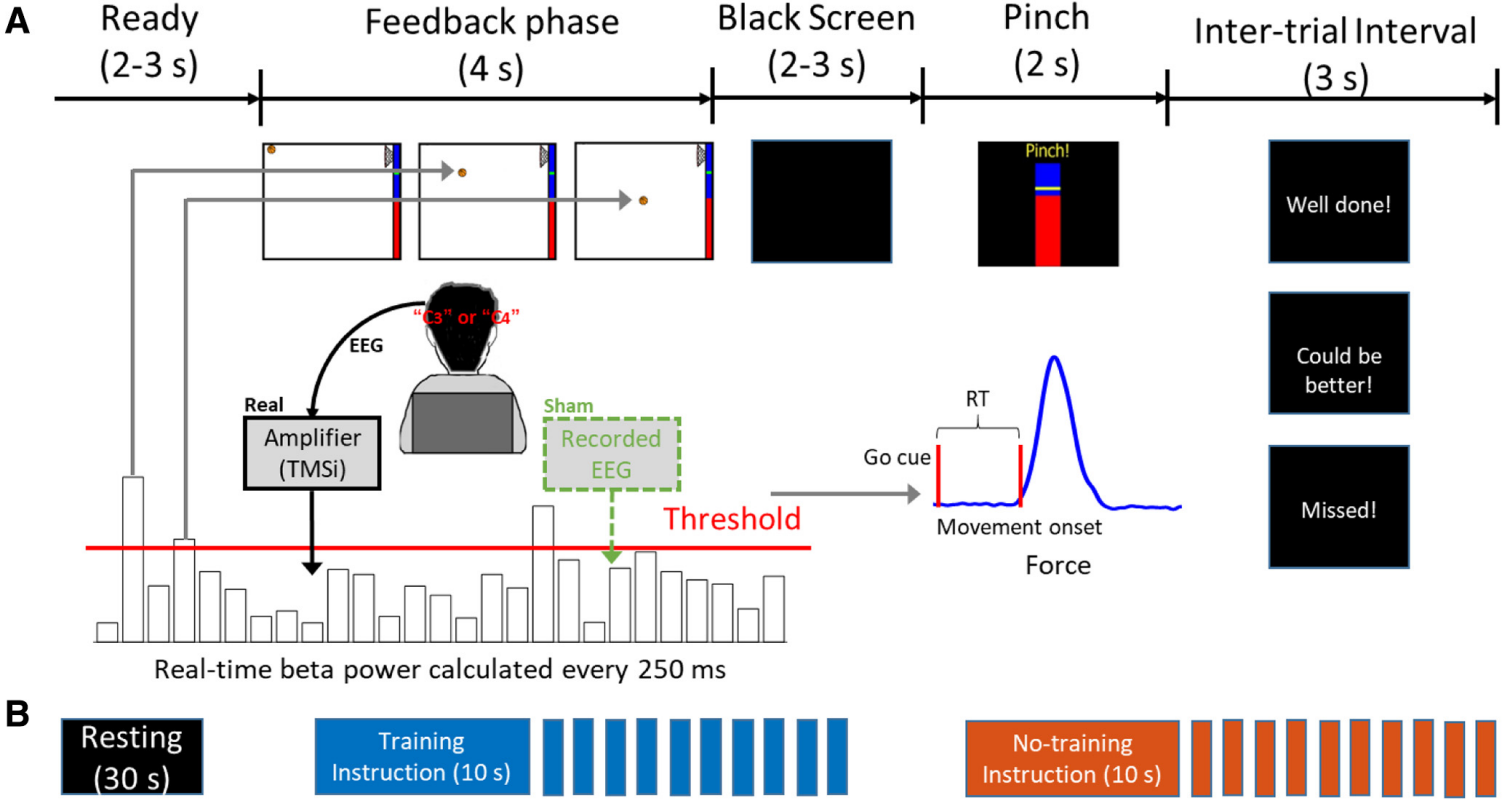
Experimental protocol. ***A***, Timeline of one individual trial. Each trial consisted of a neurofeedback phase followed by a cued pinch movement. After the finger pinch motor task, a message was displayed (“Well done!” or “Could be better!”) depending on whether the RT of the previous movement was shorter or longer than 500 ms. If movement onset was not detected within 2 s after the go-cue, the message “Missed!” was displayed. For the real feedback group, β bursts were detected in real time and used to drive the movement of the basketball in the neurofeedback phase based on EEG signals recorded from the motor cortex contralateral to the hand performing the pinch movements. For the sham feedback group, the β bursts were calculated based on a replay of EEG signals recorded from other participants. ***B***, The timeline of one experimental run which consisted of 30 s of resting, and one block of 10 continuous training trials and one block of 10 continuous no training trials. The instruction for each block was presented for 10 s before the block starts. The order of the training and no training blocks was randomized. At the beginning of each experimental run, the threshold was recalculated based on recordings made at rest.

The neurofeedback training paradigm was similar to that presented in our previous study (He et al., 2019). As shown in Figure 1, the training composed of multiple short trials. Each trial consisted of a 2-s period where the participants were instructed to get ready and a 4-s neurofeedback phase, which was followed by black screen presented for a time randomly drawn between 2 and 3 s and then a movement go-cue. The participants were instructed to perform a thumb of finger pinch movement as fast as possible in response to the go-cue to generate a force overshooting a predefined force level (50% of the maximum voluntary force measured before starting the task). Feedback about the RT of the pinch movement was provided to the participants at the end of the trial to keep participants engaged and to encourage them to be as fast as possible throughout the experiment.

During the neurofeedback phase, a basketball appeared in the top-left corner of a computer screen at the beginning of each trial (Fig. 1*A*). The position of the basketball was updated every 250 ms. The horizontal displacement for each update was constant so that the basketball moved toward the right of the screen at a constant speed during the trial. In the real feedback group, the vertical movement of the basketball was driven by the real-time detection of β bursts (when the average β power over the previous 500-ms window was over a predefined threshold) based on the EEG signal recorded from the sensorimotor cortex (C_3_ or C_4_). If a β burst was detected, the ball would drop by a fixed distance. Therefore, for the real feedback group, the final vertical position of the basketball at the end of each trial indicated how often β bursts were detected. For the sham feedback group, the vertical position of the ball determined in the same way but was driven by the β power calculated based on a replay of resting EEGs recorded from six healthy controls (aged 25–35, three females, 10-min resting for each of them) who did not participate in the main study. Specifically, at the beginning of each experiment for the sham feedback group, we randomly selected a resting EEG dataset previously recorded and randomly selected a start point within the first 5 s for the replay. Each participant completed four “training” blocks and four “no training” blocks on each recording day, with 10 trials in each block. The instruction for each block was presented for 10 s before the block starts (Fig. 1*B*). Instructions given to the participants were the same for both groups. In the training trials, the participants were instructed to keep the basketball floating at the top of the screen by preventing it from dropping. Participants were informed that motor imagery might help with the task, but they were also encouraged to try different strategies. In the no training trials, the participants were instructed to simply pay attention to the movement of the ball displayed on the screen and get ready for the subsequent movement go-cue. This design allowed us to look at the within-subjects effect of the neurofeedback training versus no training conditions and the between-subjects effect of the real feedback versus sham feedback groups. In order to reduce frustration for participants in the sham feedback group, we manually increased the threshold that triggered the drop of the basketball in the training condition by 20% so that the basketball was less likely to drop in the training condition. This manipulation was to give the participants the feeling of controlling the basketball and to reduce frustration.

Each participant was recorded for three times over three different days, with an average of 2.175 ± 0.27 d (mean ± SEM) between each recording day. On each recording day, the participant performed the neurofeedback training task with each hemisphere using the EEG signals recorded around the left/right sensorimotor cortex area, i.e., C_3_ or C_4_ (in a random order), and the contralateral hand for the motor task. Participants completed four experimental runs for each hemisphere on each training day. Each experimental run consisted of a 30 s of rest recording to calibrate the threshold for triggering the vertical movement of the basketball (see below, Calibration), 10 continuous trials in the training condition, and another 10 continuous trials in the no training condition. The order of training and no training blocks in each experimental run was randomized. In total, we recorded data from 20 hemispheres with 120 trials in each of the training and no training conditions for each hemisphere for each of the real feedback and sham feedback groups.

### Calibration

Two calibrations were performed for each tested hemisphere to: (1) select the hemisphere-specific β frequency band for the online experiment, and (2) determine the threshold *T* above which real-time quantified β power would trigger the vertical movement of the basketball in each experimental run.

First, each participant performed 30 trials of cued finger pinch movements with each hand before the experiments on the first training day. Data recorded during this task were processed offline using a continuous wavelet transform. The peak frequency (*f_p_*) in the β band (13–30 Hz) with maximal movement-related power reduction in the EEG channel located in the motor cortex (C_3_ or C_4_) contralateral to the hand performing the pinch movements was identified. Then a 5-Hz frequency band centered around *f_p_* ([*f_p_* – 2, *f_p_* + 2] Hz) was used as the hemisphere-specific β frequency band. In the current study, the peak frequencies selected for the real feedback and sham feedback groups were 19.10 ± 0.52 Hz (mean ± SEM, range between 15 and 24 Hz) and 19.20 ± 0.57 Hz (range between 15 and 24 Hz), respectively. Second, at the beginning of each experimental run, during a 30-s rest period, the average β power over each 500-ms window was estimated in real time with a 250-ms update interval, resulting in 118 β power values. Then the 75thpercentile of these values was set as the threshold *T* for the current experimental run.

### Recordings

Twenty-four EEG channels covering FP_1_, FP_2_, Fz, FCz, FC_1_, FC_2_, FC_3_, FC_4_, Cz, C_1_, C_2_, C_3_, C_4_, CPz, CP_1_, CP_2_, CP_3_, CP_4_, P_3_, Pz, P_4_, Oz, O_1_, and O_2_ according to the standard 10–20 system were recorded using a TMSi Porti amplifier (TMS International, Netherlands) at a sampling rate of 2048 Hz with a common average reference. Electromyography (EMG) signals from the flexor carpi radialis of both arms and measurements from three-dimensional accelerometers attached to the back of both hands were simultaneously recorded to monitor if the participants made any actual movements during the neurofeedback training. The generated force in the cued pinch movements was recorded using a pinch meter (Biometrics Ltd). The real time positions (*x*, *y*) of the basketball in each trial and the timing of the go-cue were also recorded and synchronized with all electrophysiological recordings through an open-source software toolkit (lab streaming layer; LSL). The paradigm used in this study was developed in C++ (Visual Studio 2017, Microsoft) and the online/offline data processing was achieved in MATLAB (R2018a, MathWorks).

After the experiment with each hemisphere on each recording day, the participants completed a NASA Task Load Index (NASA-TLX) questionnaire (Hart and Staveland, 1988). The questionnaire consists of six items on self-perceived mental demand, physical demand, temporal demand, performance, effort, and frustration level. Participants gave a score ranging between 0 and 100 to each item, with a higher score indicating a higher subjective workload. Each participant was also required to report the strategies that they used during the neurofeedback training, which were tabulated by the experimenter.

### Data analysis

#### Visual cursor movement

The trajectory of the basketball movement and the final vertical position of the basketball for each individual trial were recorded. The final vertical position was normalized to 0–100, with 0 and 100 indicating the bottom and top of the screen, respectively. The difference between the final vertical positions of the basketball between the training and no training conditions indicates the within-subjects effect of the neurofeedback training. In addition, how the difference in the basketball's final vertical positions between these two conditions varied across experimental runs and different training days indicates the learning effect with the training.

#### Motor performance

We quantified the RT of the cued pinch movement to investigate the effect of neurofeedback training on movement initiation. Force measurements from individual trials were visually inspected and those trials with movements before the go-cue and those in which the participants failed to pinch within 2 s of the imperative cue were excluded. The measured force was first bandpass filtered between 0.5–20 Hz using a fourth order zero-phase digital filter and then segmented into 4-s epochs extending between 1 s before and 3 s after the go-cue. We then calculated a threshold by taking the mean plus three times the SD of a segment of 500-ms force data before the go-cue of the pinch task. Next, the time delay between the go-cue and the time point when the force crossed the determined threshold was derived as the RT for the trial.

#### EEG β power and β bursts

The EEG from the targeted channel (C_3_ or C_4_, contralateral to the hand performing the pinch movements) was further analyzed off-line with MATLAB (v2018a, MathWorks). These signals were first bandpass filtered between 0.5–100 Hz and notch filtered at 50 Hz using a fourth order zero-phase digital filter. We then segmented these data into 10-s epochs extending from 4 s before and 6 s after the onset of the basketball movement. Time-frequency decomposition of individual trial data were obtained by continuous complex Morlet wavelet transformation with a linear frequency scale ranging from 1 to 95 Hz and a linearly spaced number (4–8) of cycles across all calculated frequencies. The time course of the average power of the selected β band was calculated for each individual trial. Average β power and two β burst characteristics (burst rate per second and accumulated burst duration) during the neurofeedback phase and during the 2-s pre-go-cue time window were calculated as described in Tinkhauser et al. (2017a) for each individual trial. This enabled comparison of average β power and burst characteristics between the training and no training conditions, and also between the real feedback group and the sham feedback group. In order to investigate whether there would be a similar impact of neurofeedback training on the bursts in other non-targeted frequency bands, we repeated the burst detection procedure and analyses in two other frequency bands by shifting the center frequency band by 8 Hz down and up, to give “β − 8Hz” and “β + 8Hz” frequency bands.

### Statistical analysis

2 × 2 ANOVAs with experimental trial conditions (training and no training) as a within group factor and feedback authenticity (real feedback and sham feedback) as a between group factor were used to evaluate the effect of the neurofeedback training conditions, feedback authenticity, and their interaction on the motor task RT and the modulation of average β power and the β burst characteristics. The assumption of sphericity was checked with Mauchly's test, and, if violated, *F* and *p* values with the Greenhouse–Geisser correction were reported. Paired and unpaired bootstrapped *t* tests (1000 permutations) were used for further *post hoc* analyses for the comparisons of within and between group measurements, respectively. Bonferroni correction was applied to adjust for multiple comparisons.

**Figure 2.**
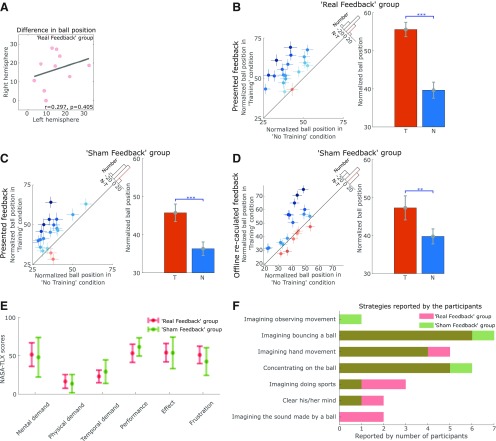
The neurofeedback training performance and the self-reported task load and strategies. ***A***, The performance of the neurofeedback control, quantified by the difference in the basketball's final positions between training and no training conditions, using the left hemisphere (C_3_) did not correlate with that using the right hemisphere (C_4_) within the same participant in the real feedback group. ***B***, In the real feedback group, participants were able to control the position of the basketball with its final position significantly higher in the training condition, *T*, compared with no training condition, N. ***C***, The differences in the basketball's final positions (presented online) between the training and no training conditions were also significant for the sham feedback group. ***D***, The basketball's final position for the training and no training conditions would also have been significantly different if they had been calculated based on the EEGs measured in real time. Results for each individual hemisphere were shown in the plots on the left, with the dots and crosses indicating the individual averages and SEMs. The shading of the dots indicates the difference between no training and training conditions, with darker blue and orange indicating higher measurement in the training and no training conditions, respectively. The *x*-axis of the histogram on the diagonal refers to the difference between no training and training conditions (N-T) and the *y*-axis refers to the number of cases. The red dotted line indicates zero. Bar plots on the right show the group averages (mean ± SEM, **p < 0.01, ***p < 0.001). ***E***, The NASA-TLX scores given by the subjects suggested that there was no difference in self-reported task load between real and sham feedback groups. ***F***, Similar strategies for controlling the ball's movement were reported by the participants in real and sham groups.

Each participant performed the task with both the left and right hemispheres and the contralateral hands separately. The targeted β frequency bands were separately calibrated for the two hemispheres, and the participants were instructed that they could have different strategies for different hemispheres. The difference in the final position of the basketball between training and no training conditions during left hemisphere-targeted neurofeedback training did not correlate with that during right hemisphere-targeted neurofeedback training (*r* = 0.297, *p* = 0.405, Pearson's linear correlation; Fig. 2*A*), suggesting that the performance of neurofeedback training in the two hemispheres within the same participant was relatively independent. Therefore, we have treated the recordings from different hemispheres as independent samples.

Linear mixed effects regression modeling (LME; implemented using the MATLAB function *fitlme*) was used to evaluate the relationship between RTs and average β power and the β bursts characteristics. The LME was also used to assess the potential learning effect across different training days. In each multilevel linear model, data from all valid individual trials in both experimental trial conditions (training and no training) from all tested hemispheres were considered. The slope(s) between the predictor(s) and the dependent variable were set to be fixed across all hemispheres; a random intercept was set to vary by hemisphere.

## Results

### The participants were able to control the position of the visual cursor driven by cortical β bursts

For the real feedback group, the basketball's final vertical position averaged across all trials over all three recording days was significantly higher in the training condition than that in the no training condition (*t*_(19)_ = 7.922, *p* = 1.939e-07, paired *t* test; Fig. 2*B*), suggesting a control of the position of the visual cursor with veritable neurofeedback training. Note that in the sham feedback group, the presented basketball's final vertical position in the training condition was also significantly higher than that in the no training condition (*t*_(19)_ = 4.940, *p* = 9.097e-05, paired *t* test; Fig. 2*C*). This was by design, and was achieved by setting a higher threshold in the training condition than the no training condition so that the basketball was less likely to drop in the training condition, to reduce frustration in the sham feedback group. Based on the recorded EEG data, we recalculated the positons of the basketball offline for the sham feedback group to see what the difference in the final basketball positions would have been if they were calculated from the EEG recorded directly from the same participants. A 2 × 2 ANOVA (ball position: “presented” and “recalculated”; condition: training and no training) on the sham feedback group identified a significant effect of condition (training vs no training: *F*_(1,38)_ = 30.54; *p* = 2.54e-06), but no effect of whether the position was presented during the main experiment or recalculated *post hoc* (*F*_(1,38)_ = 0.990; *p* = 0.326), nor significant interaction between the factors (*F*_(1,38)_ = 0.267; *p* = 0.609). As shown in Figure 2*D*, the final position of the basketball recalculated *post hoc* in the training condition was also significantly higher than no training condition (*t*_(19)_ = 3.167, *p* = 0.005, paired *t* test), suggesting that the sham feedback group did engage in various mental strategies which also reduced β bursts. The sham feedback did reflect the average β reduction due to the mental strategies but the exact timing of the basketball movements did not match the presence of β bursts. An additional 2 × 2 ANOVA (group: real feedback and sham feedback; condition: training and no training), for the ball positions (recalculated *post hoc* for the sham feedback group) revealed a significant interaction between the condition (training vs no training) and group (real feedback or sham feedback): *F*_(1,38)_ = 6.49, *p* = 0.015. This interaction suggests that the real feedback group outperformed the sham feedback group in terms of suppressing β bursts, although the sham feedback group were also able to successfully use mental strategies to reduce β bursts. To investigate whether the neurofeedback control of the basketball position was achieved by actual physical movements executed during training, we investigated the EMG and accelerometer measurements. No significant difference between the training and no training conditions was observed in the rectified EMG activities for either the real feedback (*t*_(19)_ = 0.006, *p* = 0.995, paired *t* test) or the sham feedback (*t*_(19)_ = −1.545, *p* = 0.139, paired *t* test) groups, suggesting that the neurofeedback control of the basketball position was not achieved by physical movement.

There was no significant difference between the real feedback and sham feedback groups in terms of self-reported task load such as mental demand, physical demand, performance and frustration, measured by the NASA-TLX questionnaire scores (Fig. 2*E*). Both groups reported similar strategies during the neurofeedback phase, as shown in Figure 2*F*, with imaginary movement most often reported in both groups. However other strategies, such as “concentrating on the ball,” “imaging the sound made by a ball,” and “clearing the mind” were also reported.

### Neurofeedback training led to reduced β bursts in motor cortex EEG

**Figure 3.**
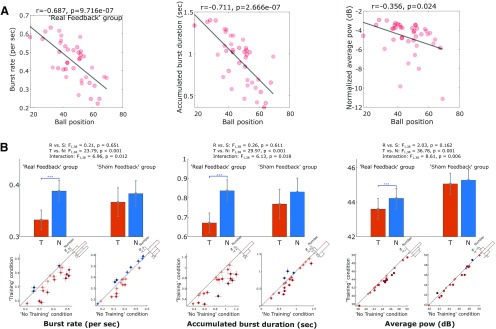
***A***, In the real feedback group, the basketball's final vertical position strongly correlated with the rate (left) and accumulated duration (middle) of β bursts, and also weakly correlated with the baseline normalized average β power during the “feedback phase.” ***B***, Burst rate (left) and accumulated burst duration (middle), as well as the average β power (right) during the neurofeedback phase were significantly reduced during training condition, *T*, compared with no training condition, N, in real feedback group, not in sham feedback group. The bar plots with error bars show the mean and SEM across tested hemisphere. The dot plots show data from individual hemispheres with the dots and crosses indicating the individual averages and SEMs. The shading of the dots indicates the difference between no training and training conditions, with darker blue and orange indicating higher measurement in the training and no training conditions, respectively. The *x*-axis of the histogram on the diagonal refers to the difference between no training and training conditions (N-T) and the *y*-axis refers to the number of cases. The red dotted line indicates zero. Bonferroni correction was applied for multiple comparisons. Values are presented as mean ± SEM; ***p < 0.001/3.

Correlational analysis with Pearson's linear correlation confirmed that for the real feedback group, the basketball's final vertical position negatively correlated with the rate of β bursts (*r* = −0.687, *p* = 9.716e-07), accumulated duration of β bursts (*r* = −0.711, *p* = 2.666e-07), and with the average β power (*r* = −0.356, *p* = 0.024) during the neurofeedback phase (Fig. 3*A*). 2 × 2 ANOVAs (condition 2: training and no training; group 2: real feedback and sham feedback) on average β power and the β burst characteristics revealed a significant main effect of condition in reducing the rate of β bursts (*F*_(1,38)_ = 23.79, *p* = 1.943e-05), the accumulated duration of β bursts (*F*_(1,38)_ = 29.97, *p* = 2.989e-06), and the average β power (*F*_(1,38)_ = 36.78, *p* = 4.641e-07) during the neurofeedback phase. There was also a significant interaction between the condition (training vs no training) and group (real feedback or sham feedback) on the rate of β bursts (*F*_(1,38)_ = 6.96, *p* = 0.012), the accumulated duration of β bursts (*F*_(1,38)_ = 6.13, *p* = 0.018), and the average β power (*F*_(1,38)_ = 8.61, *p* = 0.006). *Post hoc* analysis with paired *t* tests confirmed significant effects of the neurofeedback training in reducing the rate of β bursts (*t*_(19)_ = −4.851, *p* = 1.109e-04), the accumulated duration of β bursts (*t*_(19)_ = −5.437, *p* = 3.026e-05), and the average β power (*t*_(19)_ = −6.031, *p* = 8.407e-06), in the real feedback group, as shown in Figure 3*B*. However, there were no similar effects in the sham feedback group.

In order to investigate whether there were similar changes in other frequency bands, we repeated these tests in the β − 8Hz (mean ± SEM: 11.10 ± 0.52 Hz and 11.20 ± 0.57 Hz for the real feedback and sham feedback groups, respectively, maximum frequencies were 16 Hz for both groups) and β+8Hz (mean ± SEM: 27.10 ± 0.52 and 27.20 ± 0.57 Hz for the real feedback and sham feedback groups, respectively, maximum frequencies were 32 Hz for both groups) frequency bands. The results showed no interaction between group (real feedback and sham feedback) and condition (training and no training) on the burst characteristics and average power in the β − 8Hz frequency band, but an interaction on average power (*F*_(1,38)_ = 17.18, *p* = 0.0002) in the β+8Hz frequency band. Because of space limitation, the figures of these results are not shown. *Post hoc* analysis further showed a significant reduction of average power in the training condition compared with the no training condition in the real feedback group (*t*_(19)_ = −3.897, *p* = 9.691e-04), not in the sham feedback group (*t*_(19)_ = 1.746, *p* = 0.097) in the β+8Hz frequency band. There were no other differences: For the accumulated burst durations in the other tested frequency bands in the real feedback group (β − 8Hz: *t*_(19)_ = −2.505, *p* = 0.022; β+8Hz: *t*_(19)_ = −1.395, *p* = 0.179) or the sham feedback group (β − 8Hz: *t*_(19)_ = −1.677, *p* = 0.110; β+8Hz: *t*_(19)_ = 1.258, *p* = 0.224).

In order to evaluate whether there was any sustained carry-over effect of neurofeedback training, we repeated the abovementioned tests on the β burst characteristics and averaged β power quantified during the 2-s pre-go-cue time window when the visual feedback was no longer available. A significant interaction between the experimental condition and group was confirmed for the accumulated duration of β bursts (*F*_(1,38)_ = 4.92, *p* = 0.033), suggesting a sustained carry-over effect of neurofeedback training in reducing the accumulated β burst duration, but only for the real feedback group. No other comparisons reached significance.

### Neurofeedback training reduced RTs in subsequently cued movements

**Figure 4.**
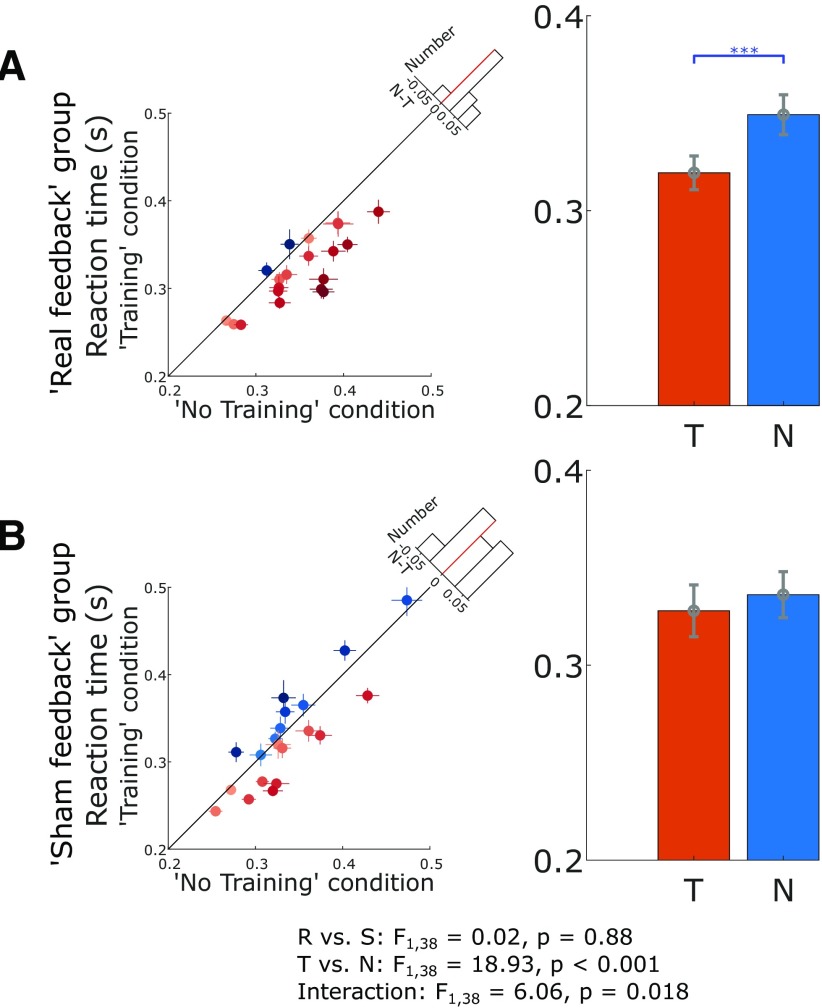
Real neurofeedback training sped up the RT in subsequently cued movement, but not sham feedback. ***A***, The RT for each individual hemisphere (left) and group-averaged RT (right) in training, *T*, and no training conditions, *N*, during real feedback. ***B***, The RT for each individual hemisphere (left) and group-averaged RT (right) in training and no training conditions during sham feedback. The dots with crosses indicate the means and cross-trial SEMs for each tested hemisphere (some data points appear to have no error bars because the lines corresponding to SEMs are shorter than the diameter of the point). The shading of the dots indicates the difference between no training and training conditions, with darker blue and orange indicating higher measurement in the training and no training conditions, respectively. The *x*-axis of the histogram on the diagonal refers to the difference between no training and training conditions (N-T) and the *y*-axis refers to the number of cases. The red dotted line indicates zero. The error bar plots show the mean and SEM across all tested hemispheres in different conditions. ***p < 0.001.

The average RT in response to the go-cue was significantly reduced in the training condition compared with the no training condition, as confirmed by a significant main effect of experimental trial condition (*F*_(1,38)_ = 18.93, *p* = 9.84e-5) in the 2 × 2 ANOVAs, which also revealed a significant interaction between condition and group (*F*_(1,38)_ = 6.06, *p* = 0.018). *Post hoc* analysis further confirmed that only real feedback group showed a significant RT reduction (0.319 ± 0.009 for the training condition compared with 0.349 ± 0.010 s for the no training condition, *t*_(19)_ = −5.089, *p* = 6.519e-05, paired *t* test; Fig. 4*A*). In contrast, there was no significant difference in the RT between the training and no training conditions for the sham feedback group (0.328 ± 0.013 compared with 0.336 ± 0.012 s, *t*_(19)_ = −1.271, *p* = 0.2191, paired *t* test; Fig. 4*B*). This was not due to different baseline levels in these two groups, as no significant difference was observed between the real feedback and sham feedback groups for RTs in the no training condition (*t*_38_ = 0.837, *p* = 0.408, un-paired *t* test).

### The RT in cued movement correlated with β burst characteristics before the go-cue

**Figure 5.**
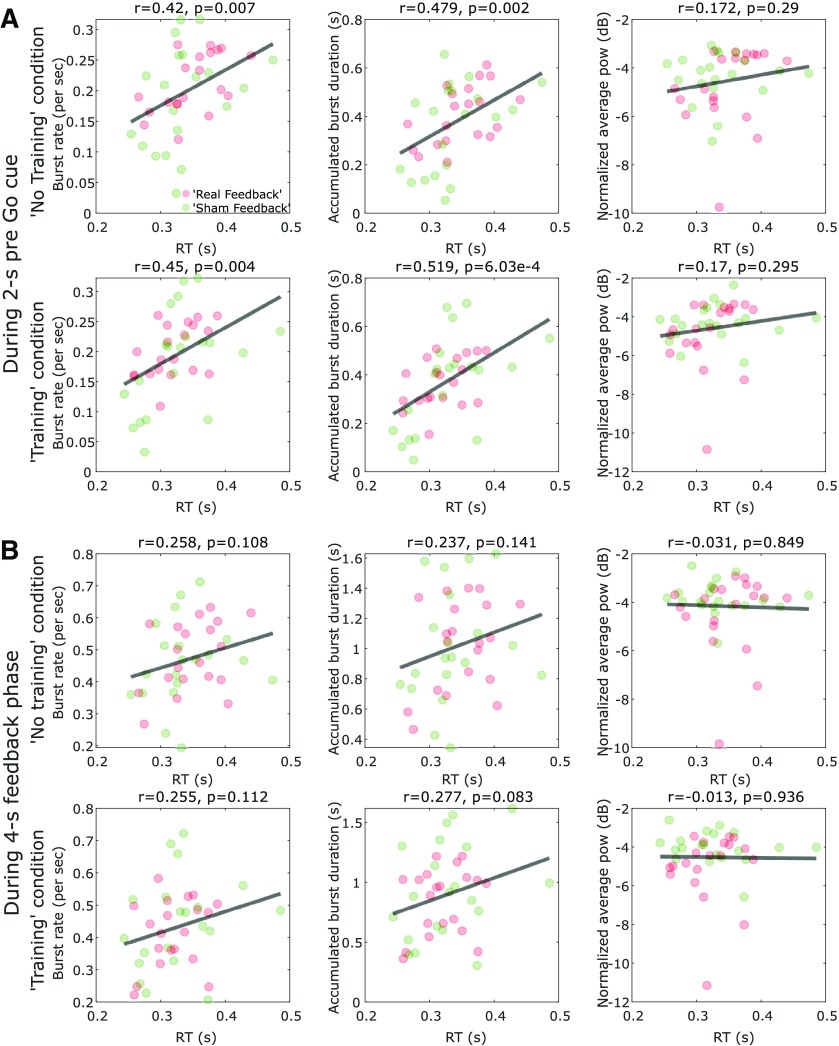
The RT of the cued pinch movements correlated with the β burst characteristics before the go-cue, but not the average β power. ***A***, There were significant positive cross-hemisphere correlations between the RT and burst characteristics during the 2 s before the go-cue in both the training and no training conditions. In contrast, there was no significant correlation between RT and average β power during the same time window. Each point indicates data from one individual hemisphere. Here the β power was baseline normalized against the average power during 30-s resting period at the beginning of each experimental run. The red and green points indicate hemispheres from the real feedback and sham feedback groups, respectively. ***B***, When the burst characteristics during the neurofeedback phase (6.5–2.5 s in average before the go-cue) were considered, the relationship with RT were positive but not significant.

When all participants in both feedback groups were considered together, there were positive significant cross-hemisphere correlations between the average RT in the subsequent cued movements and the β burst characteristics during the 2-s before the go-cue in both the no training condition (*N* = 40, *r* = 0.42, *p* = 0.007 and *r* = 0.479, *p* = 0.002 for the rate and accumulated duration of β bursts, respectively) and the training condition (*N* = 40, *r* = 0.45, *p* = 0.004 and *r* = 0.519, *p* = 6.02e-4 for the rate and accumulated duration of β bursts, respectively; Fig. 5*A*). When the β bursts characteristics during the neurofeedback phase (6.5–2.5 s in average before the go-cue) were considered, the correlations with subsequent RT were positive but not significant (shown in Fig. 5*B*).

However, the average RT did not correlate with the average β power either during the 2-s period before the go-cue (no training: *N* = 40, *r* = 0.172, *p* = 0.29; training: *N* = 40, *r* = 0.17, *p* = 0.295) or during the neurofeedback phase (no training: *N* = 40, *r* = −0.031, *p* = 0.849; training: *N* = 40, *r* = −0.013, *p* = 0.936).

LME modeling considering all individual trials was used to further assess whether there were consistent trial-to-trial correlations between RT and β characteristics within each tested hemisphere. This revealed significant within-hemisphere correlations between RTs and the accumulated duration of β bursts during the 2-s pre-go-cue phase (*k* = 0.024, *p* = 2.500e-09) and the neurofeedback phase (*k* = 0.006, *p* = 0.005), indicating that more accumulated duration of β bursts in the motor cortex before the go-cue predicts prolonged RT on trial-to-trial basis. In contrast, there was no significant effect of average β power during the neurofeedback phase or the 2-s pre-go-cue phase in predicting RT.

**Figure 6.**
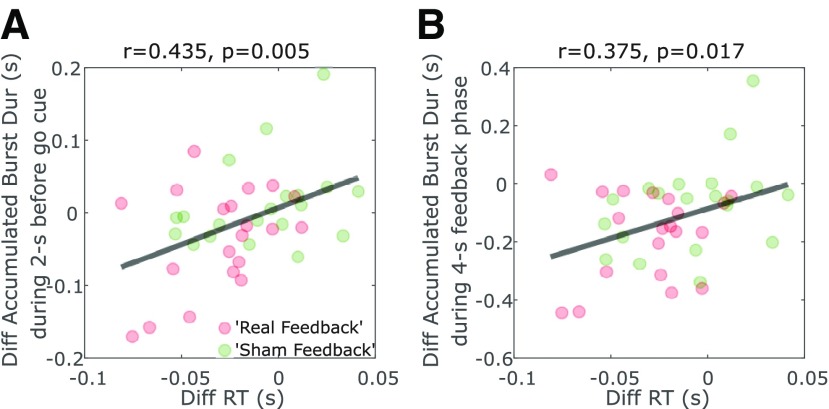
The reduced RTs in the training trials compared with no training trials positively correlated with the reduced accumulated β burst duration during the 2-s pre-cue periods (***A***) and during the 4-s “feedback phase” (***B***). Each point indicates data from one tested hemisphere. The red and green points indicate hemispheres from the real feedback and sham feedback groups, respectively.

Furthermore, the difference between RTs in the training and no training conditions significantly correlated with changes in the accumulated duration of β bursts during the 2-s pre-go-cue phase across all tested hemispheres (Pearson's linear correlation: *r* = 0.435, *p* = 0.005; Fig. 6*A*) and during the 4-s neurofeedback phase (Pearson's linear correlation: *r* = 0.375, *p* = 0.017; Fig. 6*B*). These results suggest that the hemispheres that achieved a greater reduction in β burst characteristics were associated with greater improvement in motor initiation in the contralateral hand evidenced by improved RTs. Within each hemisphere, successful β burst suppression before the go-cue predicted faster RTs on a trial-to-trial basis. In contrast, there was no significant within-hemisphere correlation between RTs and the accumulated burst duration in the β − 8Hz or β+8Hz frequency bands.

### Learning effect of neurofeedback training

**Figure 7.**
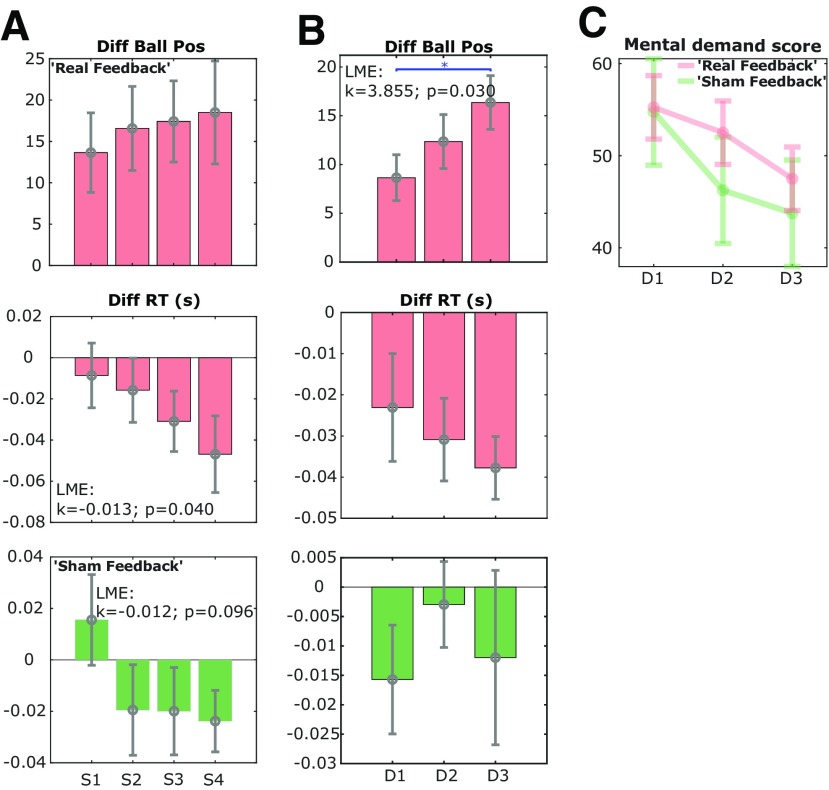
The learning effect of neurofeedback training was achieved across experimental runs and across training days. ***A***, The averaged difference in the basketball position (upper) and the RT (middle) between training and no training conditions across four experimental runs (S1–S4) on the first training day for all hemispheres in the real feedback group, and the difference in the RT for the sham feedback group (bottom). ***B***, The averaged difference in the basketball position (upper) and the RT (middle) between training and no training conditions across three training days (D1–D3) for those 75% hemispheres with the worst training effect on the first day in the real feedback group. The bottom subplot column indicates the averaged difference in RT between training and no training conditions for the 75% hemispheres with the worst training effect on the first day in the sham feedback group. ***C***, Self-reported mental demand scores decreased over the training days in both real feedback and sham feedback groups, but no significant difference was observed between these two groups. *p < 0.05.

In order to investigate whether there were any learning effects across experimental runs and training days, we quantified the difference in the basketball's final positions in the training and no training conditions for different experimental runs and different days in the real feedback group. On day 1, there was a progressive increase in the difference in the basketball position across the four experimental runs. This was accompanied by a progressive difference in the RT between the conditions (Fig. 7*A*), with a significant effect of experimental run number (*k* = −0.013, *p* = 0.040) identified by the LME modeling. There was no learning effect across different experimental runs on day 1 in the sham feedback group (*k* = −0.012, *p* = 0.096).

Across different training days, we noticed a ceiling effect in learning: by performing a quantile split for participants in the real feedback group, we observed that for the 25% hemispheres with best performance on the first day, there was no cross-day learning effects. On the other hand, for the remaining 75% hemispheres, there was significant improvement in the neurofeedback cursor control from day 1 to day 3 (*t*_(14)_ = −2.559, *p* = 0.023, paired *t* test; Fig. 7*B*), and this was accompanied by an increase in the difference in RTs over days, which was not observed in the sham feedback group. In addition, self-reported mental demand progressively reduced with training days for both real feedback and sham feedback groups, indicating another learning effect (Fig. 7*C*).

## Discussion

In this double-blind sham-controlled study, we used a sequential neurofeedback-behavior task paradigm to train healthy subjects to self-suppress β bursts in the sensorimotor cortex before a cued finger movement. The online visual feedback in the paradigm took into account the temporal dynamics of the sensorimotor β activity, and specifically targeted high amplitude β bursts. The rate of β bursts and accumulated duration of β bursts, as well as the average β power in the sensorimotor cortex were significantly reduced with real feedback training but not with sham feedback training. The real feedback group also showed a reduction in RTs in subsequent cued movements. Considering all individual trials across all tested hemispheres, the RT positively correlated with the rate and accumulated duration of β bursts before the go-cue, but not with average β power. The changes in the accumulated duration of β bursts induced by neurofeedback training positively correlated with the shortening of RT. These results are consistent with previous studies which demonstrated a positive correlation between β bursts and RT (Little et al., 2019; Wessel, 2020), but as we purposefully modulated β bursting through neurofeedback we also provide evidence in favor of a causal link between transient β bursts and motor function.

### Improved neurofeedback

Here, we show that the healthy participants gained control of the β band neural oscillations measured using EEG with the help of neurofeedback training within three training days, and the self-reported mental load progressively reduced over days. Real-time functional MRI (rtfMRI) and EEG are the most often used imaging modalities for neurofeedback training. It has been noted that EEG neurofeedback requires many sessions of training for a participant to alter electrical activity (Boulay et al., 2011; Erickson-Davis et al., 2012; McFarland et al., 2015), compared with rtfMRI in which participants can selectively modify the fMRI blood oxygen level-dependent (BOLD) signal within a few sessions of training (Subramanian et al., 2011, 2016; Thibault et al., 2016). This may be due to the lower signal-to-noise ratio in the EEG measurements and the fast time dynamics in the signal of interest. Synchrony in the β frequency band in the motor cortex or in the basal ganglia takes the form of bursts of different durations and amplitudes (Shin et al., 2017; Tinkhauser et al., 2017a,b). Directly translating continuously updated β band power into visual feedback can lead to a flickering, dynamic feedback which can be confusing for participants. Limiting the update rate, averaging data over a time window of seconds in duration, and converting continuous measurements of power into a binary visual display are often used strategies to smooth the feedback signal in EEG neurofeedback. In our paradigm, we calculated the β band power within a 500-ms window from EEG recordings and updated the visual feedback every 250 ms. This was based on previous studies showing that long β bursts (over 500 ms) are more closely related to motoric impairment in Parkinson's disease. By taking into account the temporal dynamics of the signal of interest, and reducing the variance and noise in the visual feedback that are not behaviorally relevant, our paradigm allowed healthy participants to learn to suppress β power within 30 min of training.

This is the first double-blind sham-controlled study, to our knowledge, showing that veritable neurofeedback helped participants learn to suppress cortical β bursts better than sham feedback, and accordingly, real neurofeedback training led to more behavioral benefit in speeding up movement initiation than sham feedback. Training protocols that teach people to modulate brain activities have been suggested to constitute a promising approach to motor rehabilitation for people with strokes and other disorders (Huster et al., 2014; McFarland et al., 2015).

### Sham control in neurofeedback studies

Although the sham control is important to confirm the effect of veritable neurofeedback on observed behavioral alterations compared with other mental strategies (Thibault et al., 2015, 2016), just how one should provide a proper sham control is not an easy question in neurofeedback studies. No feedback (Subramanian et al., 2011; Boe et al., 2014) or random feedback (Ramos-Murguialday et al., 2013; Ono et al., 2018) have been used as controls in other neurofeedback studies. In the current study, the feedback provided to the control group was calculated based on a replay of resting EEGs previously recorded from other healthy participants who did not participate in this study. This choice was made based on the following considerations. First, the double-blinded design was critical in this study to make sure all participants made similar effort in the task and to make sure that the study would not be biased due to the difference in the experimenter's instructions and attitude. It would have been impossible to blind the participants or the experimenter if we had provided “no feedback” to the control group, as both the participants and the experimenter would be aware of the group assignment of the participants. For similar reasons, we did not use the EEGs recorded from the real feedback group for the replay for the sham feedback group, because this would require the real feedback and sham feedback groups to be recorded in a specific order, making double-blinded design impossible. Third, without any feedback, visual attention to moving objects might induce a reduction in β band activities in the real feedback group, but not the control group (Li et al., 2015). Thus, we used the replay of previously recorded EEGs to match the dynamics in the visual feedback provided to the sham feedback and real feedback groups, which would also help control other placebo effects. Finally, real physiological EEG signals are very different compared with random signals, and thus it would be difficult to maintain similar stationarity and variability in the visual feedback for both groups if we generated feedback randomly for the control group.

It is possible that sham feedback might have interfered with the mental strategies used by the participants in the sham feedback group. We addressed this by changing the “threshold” to trigger the basketball movement so that the basketball was less likely to drop in the training condition than the no training condition. This “sham” control was necessary for the double-blinded design, as neither the experimenter nor the participants were aware of the experimental conditions. *Post hoc* analysis showed that the final position of the basketball calculated based on actual EEGs from the participants in the sham feedback group was also significantly higher in the training condition than the no training condition, suggesting that the sham feedback group did engage in various mental strategies which also reduced β bursts. In addition, the levels of β reduction in the sham feedback group were similar to those observed in other studies on β reduction during motor imagery (McFarland et al., 2000; Nam et al., 2011). There was no significant difference between the sham feedback and real feedback groups in terms of self-reported mental effort as well as mental strategies used.

*Post hoc* analysis showed that the average basketball positions presented during the experiments and those recalculated offline were similar in the sham feedback group. This means that the sham feedback did reflect the average β reduction due to the mental strategies employed by participants in the sham feedback group. The main difference between the real feedback and sham feedback was whether the exact timing of the basketball movements matched with the presence of the β bursts. Our reported interaction between the group (real feedback vs sham feedback) and experimental condition (training vs no training) confirmed that veritable real-time neurofeedback had additional effects in training the participants to learn the “skill” of suppressing β bursts. This is consistent with our results in Figure 7*A*,*B* showing that only in the real feedback group did performance improve across experimental runs and across training days. More importantly, real neurofeedback training led to a behavioral benefit in speeding up movement initiation more than sham feedback, suggesting an additional benefit of the real feedback compared with the sham feedback.

### Is β bursts or average β power related to motor performance?

Movement-related power reduction before and during voluntary movement in the β frequency band has been consistently observed at different sites in the cortical-basal ganglia motor network in both healthy subjects (Pfurtscheller and Lopes da Silva, 1999; Tan et al., 2014a; Torrecillos et al., 2015) and patients with Parkinson's disease (Cassidy et al., 2002; Doyle et al., 2005; Tan et al., 2014b). In particular, Kühn et al. (2004) found that the event-related desynchronization (ERD) of β power in the human subthalamic nucleus correlated with motor performance, with a longer latency ERD associated with longer RTs in an externally paced voluntary movement. Such movement-related β power reduction has been suggested to reflect changes in the probability of β bursts rather than a smooth modulation of sustained β activity (Feingold et al., 2015), and more recently, it has been emphasized that the β bursts in the cortical-basal ganglia motor network predict sensorimotor behavior (Feingold et al., 2015; Sherman et al., 2016; Shin et al., 2017; Torrecillos et al., 2018; Little et al., 2019; Wessel, 2020) and correlate with motor impairment in patients with Parkinson's disease (Tinkhauser et al., 2017a,b). Indeed, β bursts in the subthalamic nucleus, a key relay in basal ganglia-cortical circuits, are more predictive of motor impairment than average β power in patients with Parkinson's disease (Torrecillos et al., 2018; Tinkhauser et al., 2020), and similar observations have been made regarding cortical β bursts and trial-averaged β power. Little et al. (2019) showed that the timing of β bursts was a stronger predictor of single trial behavior than single trial-averaged β amplitude. Here we also show that the RT of cued movements correlates with the incidence of pre-movement β bursts, and not with the average β power. In addition, we show that healthy participants can volitionally modulate the incidence of β bursts with neurofeedback training, and this as associated with improvement in motor initiation, raising the possibility that the more direct link between motor behavior and β activity is with β bursts rather than averaged levels of β power.

The current study therefore strengthens the link between movement initiation and pre-movement β bursts in the sensorimotor cortex; and also helps establish an association between neurofeedback training and enhanced performance in terms of both self-regulation of pre-movement β bursts and movement initiation. Thus, this study emphasizes the importance of better characterizing the temporal dynamics of the targeted brain activity when using neurofeedback training to change behavior.

### Detecting β bursts in real time and other limitations

In this study, we used a threshold technique applied to the average β power over the last 500-ms time window to define the presence or absence of β bursts in real time. In order to take into account any potential drift in baseline β activity, we quantified the threshold as the 75th percentile value of the averaged β power over 500-ms time windows recorded while participants were at rest before each experimental run. Cortical β bursts are reported to have shorter durations than the 500-ms window used in our real-time analysis (Sherman et al., 2016; Shin et al., 2017). Nevertheless the incidence of the “β bursts” detected in real time, indicated by the vertical position of the visual cursor use in the paradigm correlated well with the β burst rate quantified in postprocessing (Fig. 3*A*), with the vertical position of the visual cursor capturing around 50% of the variance in β burst characteristics revealed by postprocessing using more conventional burst-identifying techniques, such as those reported by Tinkhauser et al. (2017a,b). The 500-ms time window for quantifying β power in real time used here afforded a compromise whereby excessive flickering was avoided but sensitivity of neurofeedback was maintained. In the future it would be worthwhile investigating whether other β burst-detection methods might be more efficient in real time.

Another limit of the study is: the current paradigm aims to give feedback about the occurrence of β bursts, therefore, the visual cursor (the basketball displayed on the screen) can only drop down once a β burst is detected but cannot rise up again. Thus, the visual cursor might have already dropped to the bottom of the screen before the end of the trial, making the participants stop trying before the end of trial. The neurofeedback paradigm may be further improved by allowing the visual cursor to rise again if β bursts were suppressed successfully.

## Summary

In this double-blind sham-controlled EEG neurofeedback study, we showed that veritable neurofeedback training can help participants learn to suppress β bursts in motor cortex, and this was associated with speeding up in movement initiation in response to a subsequent go-cue. The ability of β burst self-modulation was significantly higher with veritable feedback than with sham feedback, and the improvement in movement initiation was only observed with veritable feedback. These results provide evidence of a causal link between pre-movement β bursts and delayed movement initiation. The study also suggests neurofeedback training targeting β bursts may be an effective approach to improve movement initiation.
